# Tau accumulation is associated with dopamine deficiency in vivo in four-repeat tauopathies

**DOI:** 10.1007/s00259-024-06637-6

**Published:** 2024-02-17

**Authors:** Christian Ferschmann, Konstantin Messerschmidt, Johannes Gnörich, Henryk Barthel, Ken Marek, Carla Palleis, Sabrina Katzdobler, Anna Stockbauer, Urban Fietzek, Anika Finze, Gloria Biechele, Jost-Julian Rumpf, Dorothee Saur, Matthias L. Schroeter, Michael Rullmann, Leonie Beyer, Florian Eckenweber, Stephan Wall, Andreas Schildan, Marianne Patt, Andrew Stephens, Joseph Classen, Peter Bartenstein, John Seibyl, Nicolai Franzmeier, Johannes Levin, Günter U. Höglinger, Osama Sabri, Matthias Brendel, Maximilian Scheifele, for the German Imaging Initiative for Tauopathies (GII4T)

**Affiliations:** 1 Department of Nuclear Medicine, LMU University Hospital, LMU Munich, Munich, Germany; 2 Department of Nuclear Medicine, University Hospital Leipzig, Leipzig, Germany; 3 InviCRO, LLC, Boston, MA USA; 4 Molecular Neuroimaging, A Division of inviCRO, New Haven, CT USA; 5 Munich Cluster for Systems Neurology (SyNergy), Munich, Germany; 6 German Center for Neurodegenerative Diseases (DZNE), Munich, Germany; 7 Department of Neurology, LMU University Hospital, LMU Munich, Munich, Germany; 8 Department of Radiology, LMU University Hospital, LMU Munich, Munich, Germany; 9 Department of Neurology, University Hospital Leipzig, Leipzig, Germany; 10 Clinic for Cognitive Neurology, University Hospital Leipzig, Leipzig, Germany; 11 LIFE - Leipzig Research Center for Civilization Diseases, University of Leipzig, Leipzig, Germany; 12 Max Planck Institute for Human Cognitive and Brain Sciences, Leipzig, Germany; 13 Life Molecular Imaging GmbH, Berlin, Germany; 14 Institute for Stroke and Dementia Research, LMU University Hospital, LMU Munich, Munich, Germany

**Keywords:** 4R-Tau, DaT imaging, [^18^F]PI-2620 tau-PET, Motor reserve

## Abstract

**Purpose:**

We hypothesized that severe tau burden in brain regions involved in direct or indirect pathways of the basal ganglia correlate with more severe striatal dopamine deficiency in four-repeat (4R) tauopathies. Therefore, we correlated [^18^F]PI-2620 tau-positron-emission-tomography (PET) imaging with [^123^I]-Ioflupane single-photon-emission-computed tomography (SPECT) for dopamine transporter (DaT) availability.

**Methods:**

Thirty-eight patients with clinically diagnosed 4R-tauopathies (21 male; 69.0 ± 8.5 years) and 15 patients with clinically diagnosed α-synucleinopathies (8 male; 66.1 ± 10.3 years) who underwent [^18^F]PI-2620 tau-PET and DaT-SPECT imaging with a time gap of 3 ± 5 months were evaluated. Regional Tau-PET signals and DaT availability as well as their principal components were correlated in patients with 4R-tauopathies and α-synucleinopathies. Both biomarkers and the residuals of their association were correlated with clinical severity scores in 4R-tauopathies.

**Results:**

In patients with 4R-tauopathies, [^18^F]PI-2620 binding in basal ganglia and midbrain regions was negatively associated with striatal DaT availability (i.e. globus pallidus internus and putamen (β =  − 0.464, *p* = 0.006, Durbin-Watson statistics = 1.824) in a multiple regression model. Contrarily, [^18^F]PI-2620 binding in the dentate nucleus showed no significant regression factor with DaT availability in the striatum (β = 0.078, *p* = 0.662, Durbin-Watson statistics = 1.686). Patients with α-synucleinopathies did not indicate any regional associations between [^18^F]PI-2620-binding and DaT availability. Higher DaT-SPECT binding relative to tau burden was associated with better clinical performance (β =  − 0.522, *p* = 0.011, Durbin-Watson statistics = 2.663) in patients with 4R-tauopathies.

**Conclusion:**

Tau burden in brain regions involved in dopaminergic pathways is associated with aggravated dopaminergic dysfunction in patients with clinically diagnosed primary tauopathies. The ability to sustain dopamine transmission despite tau accumulation may preserve motor function.

**Supplementary Information:**

The online version contains supplementary material available at 10.1007/s00259-024-06637-6.

## Background

Progressive supranuclear palsy (PSP) and corticobasal degeneration (CBD) are primary tauopathies that belong to the spectrum of atypical parkinsonian syndromes. While neurodegenerative parkinsonian syndromes (i.e. Parkinson’s disease (PD) and atypical parkinsonian disorders) jointly show a loss of function in the dopaminergic system, PSP and CBD are histopathologically distinct compared to α-synucleinopathies such as PD and multiple systems atrophy (MSA). The key histopathological features in PSP and CBD are pathological neuronal and glial cell inclusions of the four-repeat (4R)-tau isoform [1–3]. 4R-tau pathology plays a key role in neuronal dysfunction and its accumulation in PSP and CBD is thought to follow specific spatiotemporal patterns, initially accumulating in the brainstem and subcortical areas followed by cortical deposition in later disease stages [4–7]. There is growing evidence that the spreading of tau in neurodegenerative disorders occurs in a prion-like manner into anatomically and functionally connected regions of the brain thereby enabling disease progression [8–12]. Yet the connection between pathological tau accumulation and loss of dopaminergic cells in 4R-tauopathies remains poorly understood. In this study, we aimed to elucidate the association between tau pathology and dopaminergic loss using [^18^F]PI-2620 tau- positron-emission-tomography (PET) and [^123^I]-Ioflupane single-photon-emission-computed tomography (SPECT) imaging in the same individuals in vivo. Using PET radiotracers, imaging of tau deposits has become feasible lately and second generation tau-PET tracers such as [^18^F]PI-2620 [13] and [^18^F]PM-PBB3 [14] provide new possibilities to detect not only 3/4R-tau depositions typically found in Alzheimer’s disease (AD) but also have affinity to 4R-tau. Our consortium showed autoradiography binding to PSP tissue in vitro and discrimination of patients with PSP and corticobasal syndrome (CBS) from controls in vivo using [^18^F]PI-2620 [15–18]. Others were successful to show in vitro binding and differentiation of 4R-taupathies from controls in vivo using [^18^F]PM-PBB3 [14, 19, 20]. [^123^I]-Ioflupane on the other hand is a well-established SPECT ligand for in vivo imaging of the dopaminergic system with a high affinity to the striatal presynaptic dopamine transporter (DaT)[21]. In a clinical setting, [^123^I]-Ioflupane SPECT imaging is frequently used to differentiate degenerative parkinsonian disorders from non-degenerative parkinsonism (e.g. vascular, toxic, drug-induced) as well as to distinguish Dementia with Lewy bodies (DLB) from AD [22, 23]. Using [^18^F]PI-2620 tau-PET, we hypothesized that high tau burden in brain regions involved in direct or indirect pathways of the basal ganglia correlates with a more severe loss of DaT availability visualized by [^123^I]-Ioflupane SPECT imaging.

## Methods

### Study design and patient selection

Thirty-eight patients with a clinically diagnosed 4R-tauopathy [PSP with Richardson syndrome (PSP-RS) and CBD with corticobasal syndrome phenotype (CBD-CBS)] were examined in comparison to a group of 15 patients with assumed α-synucleinopathies [Parkinson’s disease (PD), multiple systems atrophy (MSA), DLB] in this combined tau-PET and DaT-SPECT study.

Patients with 4R-tauopathies according to current diagnostic criteria [24] were recruited from three different centers: Munich, Leipzig and New Haven (25 cases from the LMU University Hospital of Munich, 9 cases from Leipzig and 4 cases from New Haven). The 4R-tauopathy group (age: 69.0 ± 8.5 years, 21 male) consisted of 26 patients with PSP-RS (PSP rating scale: 36.9 ± 13.8), 12 fulfilling criteria for CBD-CBS (PSP rating scale: 36.0 ± 11.7). Patients with clinically diagnosed α-synucleinopathies (age: 66.1 ± 10.3 years, 8 male) consisted of 13 cases from Munich and two cases from Leipzig, characterized by 10 patients with PD, three patients with MSA, two patients with DLB. Both groups were evaluated by dual imaging of [^18^F]PI-2620 tau-PET and [^123^I]-Ioflupane SPECT. The two scans were performed with a time gap of 3 ± 5 months and a maximum allowed time gap of 2 years.

To quantify the tau-PET scans, z-scores were calculated against an age-matched cognitively healthy control group consisting of 23 individuals from three different centers in Munich (*n* = 10), New Haven (*n* = 8) and Melbourne (*n* = 5).

Patients from Munich are part of the observational study registered at the German Clinical Trials Register (DRKS00016920) and the tau-PET data from these patients were partly published elsewhere [15, 18]. All participants provided written informed consent for PET imaging. The study protocol as well as PET data analyses were approved by the local ethics committee of LMU of Munich (application numbers 17–569 and 19–022). The study was carried out according to the principles of the Helsinki Declaration.

### [^18^F]PI-2620 tau-PET and [^123^I]-Ioflupane DaT-SPECT imaging

#### Radiosynthesis

Radiosynthesis of [^18^F]PI-2620 was achieved by nucleophilic substitution on a BOC-protected nitro precursor using an automated synthesis module (IBA Synthera, Louvain-la-neuve, Belgium). The protecting group was cleaved under the radiolabelling conditions. The product was purified by semipreparative HPLC. Radiochemical prurity was 99%. Non-decay yields were about 35% with a molar activity of 3•10^6^ GBq/mmol at the end of synthesis. [^123^]-Ioflupane was purchased from GE healthcare.

### Dynamic [^18^F]PI-2620 tau-PET acquisition and reconstruction

The cohort of this study underwent scanning in a full dynamic setting (0–60 min p.i.) on two different scanners at the Department of Nuclear Medicine, LMU Munich, either a Biograph 64 or a Siemens mCT PET/CT scanner (both Siemens, Erlangen, Germany), on a Siemens ECAT EXACT HR + camera at MNI, on a Siemens Biograph mMR (Siemens, Erlangen, Germany) in Leipzig and on a Philips Gemini TF 64 PET/CT (Eindhoven, The Netherlands) in Melbourne. Details on all scanners, as well as acquisition and reconstruction parameter are provided in the Supplement of our previous study [15]. The intravenous injected bolus dose was 168 to 334 MBq and was followed by a 10 ml saline flush [25]. Continuous brain imaging started right after the injection, divided into a series of 23 frames (6 × 30 s, 4 × 60 s, 4 × 120 s, and 9 × 300 s). Before processing, all dynamic images underwent correction for head motion or non-standard posture (i.e. excessive head hypokinesis), in case they did not pass a visual check. A single 20–40 min frame was summed and analyzed after motion correction [25].

### DaT-SPECT image acquisition and reconstruction

In order to protect against irradiation of the thyroid, the subjects were pretreated with perchlorate at 30–60 min before intravenous injection of a mean 145 ± 7 MBq [^123^I]-Ioflupane as a single bolus. Single frame SPECT emission recordings of 30 min duration were recorded at four hours after tracer injection using a triple-headed gamma camera (Picker Prism3000, Cleveland, OH) equipped with low-energy, high-resolution fan beam collimators. The acquisition parameters consisted of a rotational radius of 12.7–13.0 cm, a 20%energy window centered on 159 keV, 120 projection angles over 360° (60 s per projection), and a 128 × 128 matrix. Images were reconstructed by filtered back- projection (low-pass filter with a cut-off frequency of 0.27Nyquist, fifth order) and corrected for attenuation according to Chang (µ = 0.12 cm^−1^). Final voxel size in the reconstructed and reoriented images was 2.26 × 2.26 × 3.56 mm.

### Tau-PET data analysis

All image data were processed and analyzed with PMOD (Version 3.4, PMOD Technologies Ltd., Zurich, Switzerland). Late phase 20–40 min [^18^F]PI-2620 PET images were coregistered to the Montreal Neurology Institute (MNI) space using a non-linear transformation (brain normalization settings: nonlinear warping, 8 mm input smoothing, equal modality, 16 iterations, frequency cutoff 3, regularization 1.0, no thresholding) [26]. Standardized uptake value ratios (SUVr) were generated by dividing the 20–40 min static [^18^F]PI-2620 PET images through a cerebellar reference region, excluding the dentate nucleus, the cerebellar white matter and superior and posterior layers [15]. SUVr were extracted from 13 PSP target regions of interest in the MNI space: bilateral putamen, bilateral globus pallidus (internal and external part), bilateral subthalamic nucleus, bilateral substantia nigra, dorsal midbrain, bilateral dentate nucleus while keeping in mind that substantia nigra generally shows a higher background signal possibly due to additional neuromelanin off-target binding [15]. Tau-PET z-scores were calculated against the age-matched cognitively healthy control group.

### DaT-SPECT data analysis

The DaT-SPECT scans were examined as DICOM files by using Hermes BRASS (Hermes Medical Solutions, Stockholm, Sweden) with the occipital lobe as the reference region (BRASS model 5 and BRASS model 7). Caudate nucleus, anterior putamen and posterior putamen served as target regions (all bilateral) and z-scores against healthy controls with correction for age were calculated [27]. DaT-SPECT data of one patient from New Haven had to be evaluated with manual created VOIs fitting to the selected areas by using PMOD (Version 3.4, PMOD Technologies Ltd., Zurich, Switzerland) due to failed coregistration to the template of the software package.

### Statistics

The statistical analyses were performed using SPSS (version 26.0, Armonk, New York, USA) and Excel (Microsoft, Redmond, WA, USA). Statistical significance was defined at a level of *p* < 0.05 in all analyses. The data showed as n ± x represent the average and the standard deviation. Tau-PET and DaT-SPECT z-scores were normally distributed, as assessed by the Kolmogorov–Smirnov-test, *p* > 0.05. *T*-tests and X^2^ tests were performed to compare demographics between patients with 4R-tauopathies and patients with α-synucleinopathies. One-way analysis of variance with age, gender and center as covariates (ANCOVA) was performed for comparison of tau-PET and DaT-SPECT SUVr and z-scores including *p* value and η^2^ as a measure of effect size.

We calculated a multiple regression analysis including the two biomarkers as well as age, gender and center as covariates in all target regions. The Durbin-Watson statistics of all regression models performed in this study provided a value close to 2.0, which showed that the residuals were independent and not inter-correlated. We performed a principal component analysis (PCA) for each of the two biomarkers concerning all target regions in order to achieve a dimensional reduction of the data to its essential features while mitigating possible effects of multileg testing. Subsequently, we performed a multiple regression analysis including the calculated components. The Kaiser-Mayer-Olkin (KMO) measure for sampling adequacy and Bartlett’s test of sphericity showed that the data was suitable for data-driven dimension reduction.

To assess potential motor reserve effects, we performed a multiple regression analysis including the residuals of the Tau/DaT regression model and PSP rating scale scores. Moreover, the PSP rating scale was differentiated between items that cover motor and cognition function, which then were included in a separate multiple regression model.

## Results

### Demographics

A total of 38 patients with 4R-tauopathies and 15 patients with α-synucleinopathies were included in the analysis (Supplementary Table 1). Patients with 4R-tauopathies did not differ in age (69.0 ± 8.5 years vs. 66.1 ± 10.3 years; *p* = 0.21; *t*-test) and gender (55% male vs. 53% male; *p* = 0.899; X^2^ test) from patients with α-synucleinopathies. Patients with 4R-tauopathies showed a mean disease duration of 26.2 ± 16.6 months compared to a mean disease duration of 15.6 ± 10.6 months in patients with α-synucleinopathies (*p* = 0.27; *t*-test).

### [^18^F]PI-2620 tau-PET and [^123^I]-Ioflupane DaT-SPECT binding

**Fig. 1:**
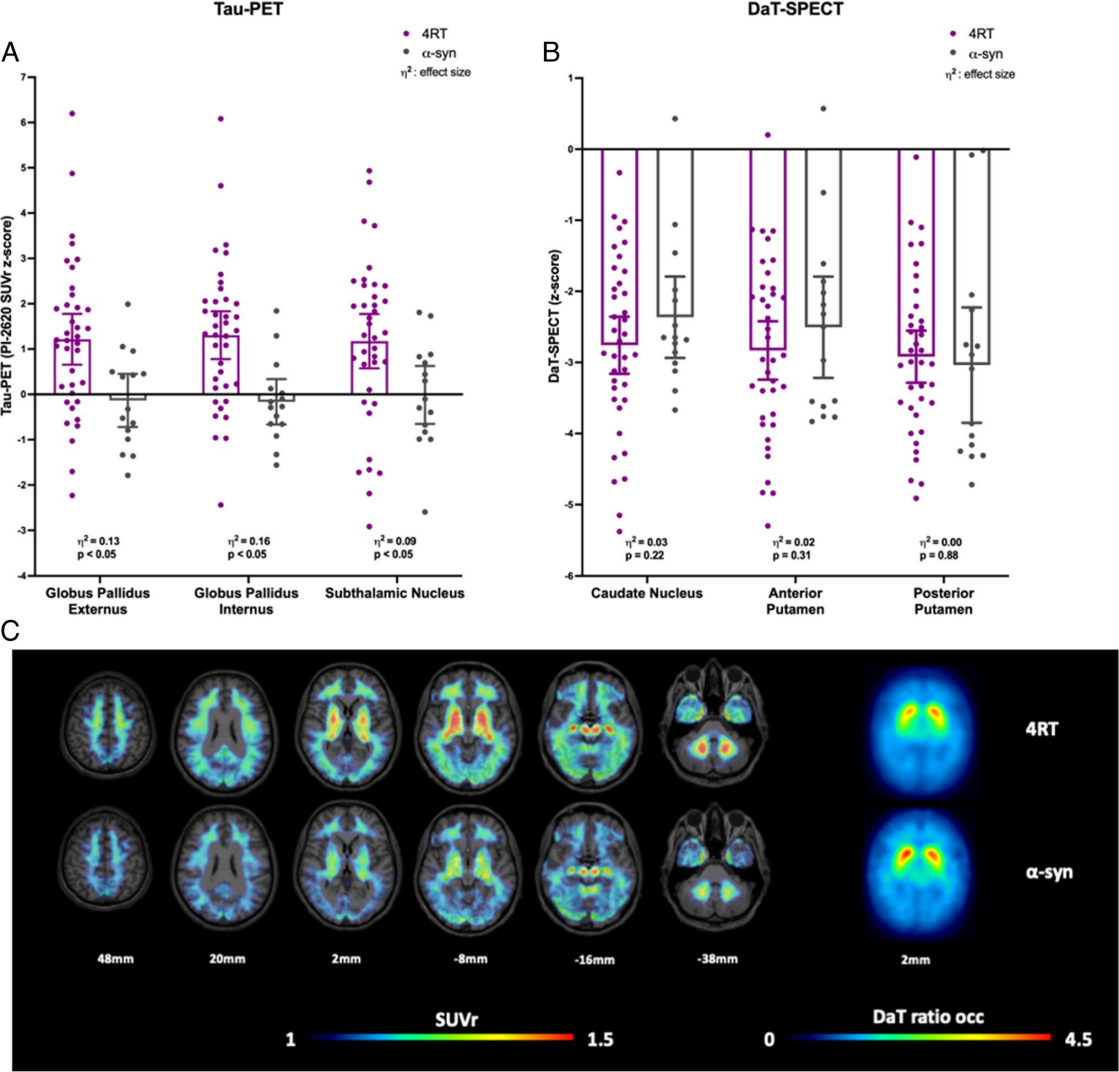
Quantitative tau-PET and DaT-SPECT comparison of patients with clinical diagnosis of 4R-tauopathies (4RT) and α-synucleinopathies (α-syn). **A** Z-score distribution of tau-PET including p-value and effect size η^2^ in representative brain regions of patients with 4R-tauopathies and patients with α-synucleopathies. SUVr = standardized uptake value ratio. **B** Z-score distribution of DaT-SPECT including p-value and effect size η^2^ in comparison of patients with 4R-tauopathies and patients with α-synucleopathies. **C** Tau-PET SUVr and DaT-SPECT ratio images show the group average of patients with clinically diagnosed 4R-tauopathies and clinically diagnosed α-synucleinopathies

As expected from previous studies [15, 18], higher [^18^F]PI-2620 signal was observed all 13 target regions of patients with 4R-tauopathies when compared to patients with α-synucleinopathies (Fig. 1a, Supplementary Table 2). Strongest regional [^18^F]PI-2620 signal differences between patients with 4R-tauopathies and patients with α-synucleinopathies were observed in the right globus pallidus internus (SUVr: 1.47 ± 0.27 vs 1.27 ± 0.14; *p* = 0.028, ANCOVA controlled for age, gender and center). 63.2% of patients with 4R-tauopathies and 6.7% of patients with α-synucleinopathies (*p* < 0.001; X^2^ test) showed at least one [^18^F]PI-2620 positive target region (z-score > 2). In regard to the SN the right SN showed a significantly higher signal in 4R patients than in α-synucleinopathies while there was no significant difference to HC (SUVr: 1.36 ± 0.20 vs 1.24 ± 0.11 α-syn vs 1.32 ± 0.12 HC; p(α-syn): 0.04, p(HC): 0.50; ANCOVA controlled for age, gender and center). Quantitative DaT-SPECT showed similar dopamine deficiency in patients with 4R-tauopathies compared to patients with α-synucleinopathies (Fig. 1b, Supplementary Table 3). DaT-SPECT binding in at least one target region was significantly reduced (z-score <  − 2) in 89.5% of patients with 4R-tauopathies and in 86.7% of patients with α-synucleinopathies (*p* < 0.001; X^2^ test). Figure 1c visualizes Tau-PET SUVr and DaT-SPECT ratio images of the group average of patients with 4R-tauopathies versus α-synucleinopathies.

### Regional associations between tau burden and DaT availability

**Fig. 2:**
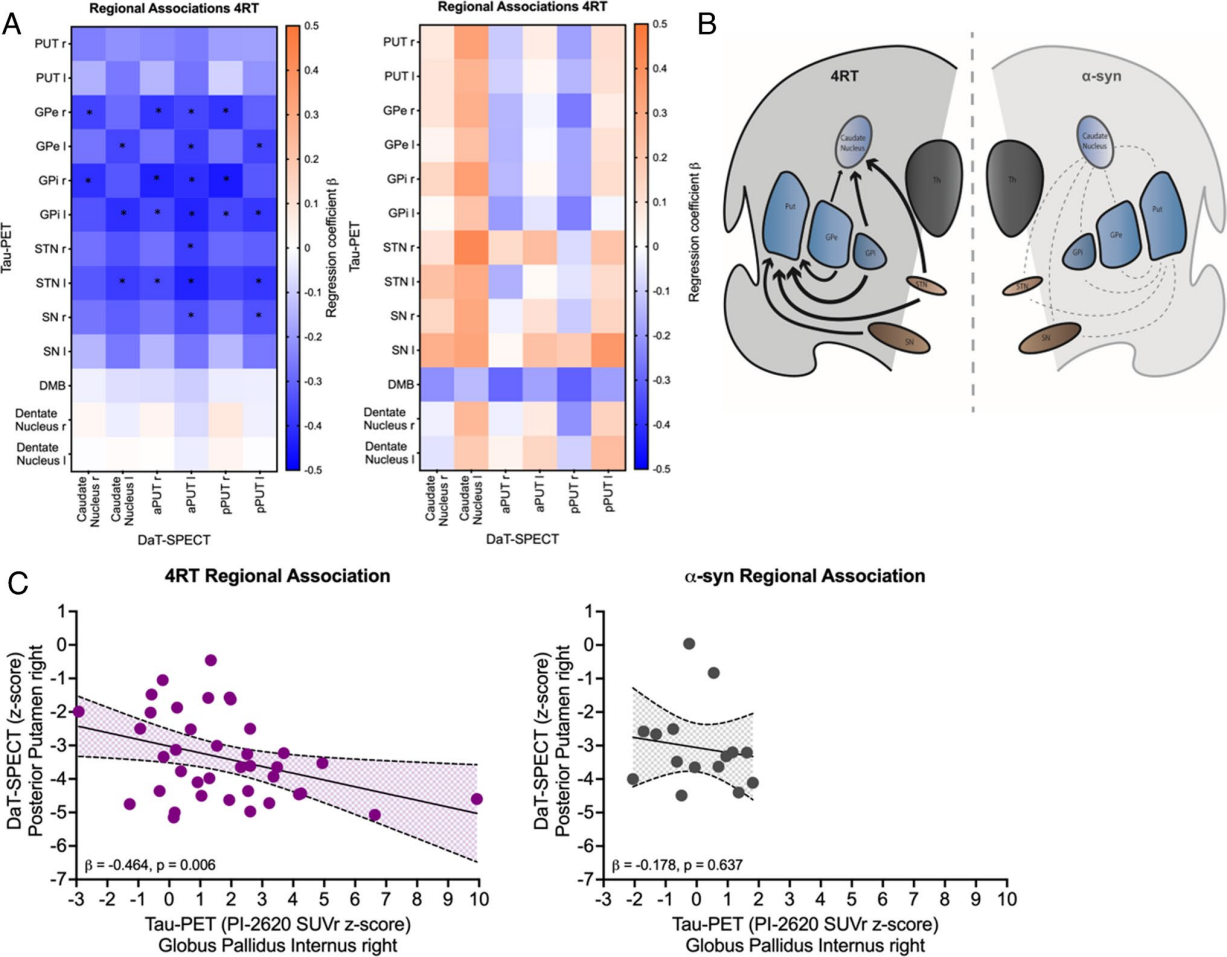
Regional associations between tau-PET and DaT-SPECT in patients with 4R-tauopathies (4RT) and α-synucleinopathies (α-syn). **A** Heat maps show regional associations between tau-PET (y-axis) and DaT-SPECT (x-axis). Significant associations providing a p value < 0.05 are indicated with *. Blue colors indicate negative multiple regression coefficients (β). Orange colors indicate positive multiple regression coefficients (β). Regions analyzed for tau-PET: putamen right, left (PUT r/l), globus pallidus externus right, left (GPe r/l), globus pallidus internus right, left (GPi r/l), subthalamic nucleus right, left (STN r/l), substantia nigra (SN r/l), dorsal midbrain (DMB) and dentate nucleus right, left (Dentate r/l). Regions analyzed for DaT-SPECT: caudate right, left, anterior putamen right, left (aPUT r/l) and posterior putamen right, left (pPUT r/l). **B** The scheme shows the basic idea of the connection between high tau burden in brain regions involved in direct or indirect pathways of the basal ganglia and striatal dopaminergic loss. Arrow thickness shows the level of correlation of the examined regions for patients with 4R-tauopathies, whereas no significant correlations were found in α-synucleopathies (dashed lines). **C** Linear correlation between tau burden in the globus pallidus internus and the DaT availability in the posterior putamen of the right hemisphere

In patients with 4R-tauopathies, several negative associations were observed between regional tau-PET signal and DaT-SPECT binding in the basal ganglia in a multiple regressions model including age, gender and center (Fig. 2a). Contrary, there was no significant association between tau-PET and DaT-SPECT quantification in patients with α-synucleinopathies (Fig. 2a). As an example, tau-PET signal in the right globus pallidus internus showed the strongest negative regression factor with DaT availability in the right posterior putamen of patients with 4R-tauopathies (β =  − 0.464, *p* = 0.006, Durbin-Watson statistics = 1.824; Fig. 2c), whereas this association was not observed in patients with α-synucleinopathies (β =  − 0.178, *p* = 0.637, Durbin-Watson statistics = 1.810; Fig. 2c). As a negative control region without striatal projection, tau-PET binding in the dentate nucleus was not associated with DaT-SPECT loss in both patient populations (Fig. 2a). Due to asymmetrical and higher DaT loss on the left hemisphere in our patient group, we also examined regional associations focused on the more affected side of each patient, which showed a similar negative association (Supplementary Fig. 1). A schematic visualization of the connection between brain regions with high tau burden and striatal dopaminergic loss is given in Fig. 2b.

### Data-driven tau-PET and DaT-SPECT correlation

**Fig. 3:**
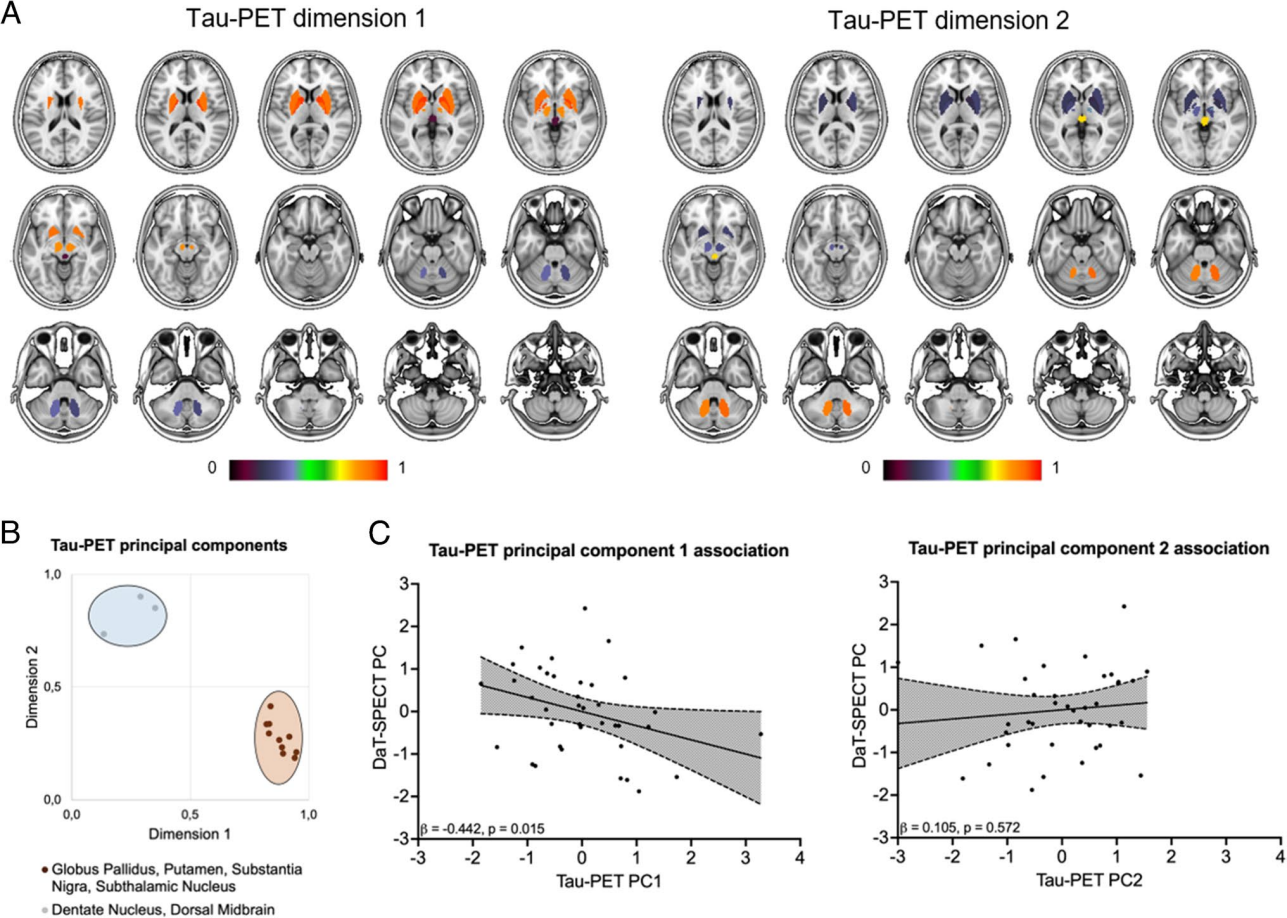
Data driven correlation between tau burden and DaT availability. **A** Visualization of the brain regions resulting from the principal component analysis of tau-PET target regions. The color bar indicates corresponding loading values. Two principal components emerged, consisting of putamen, globus pallidus, subthalamic nucleus and the substantia nigra (principal component 1) as well as dentate nucleus and dorsal midbrain (principal component 2). **B** Visualized values of the rotated component matrix derived from the principal component analysis. **C** Tau-PET principal component 1 (tau-PET PC1) indicated a significant negative association with the DaT-SPECT principal component (DaT-SPECT PC), whereas tau-PET principal component 2 (tau-PET PC2) was not associated with the DaT-SPECT PC in a multiple regression model

To achieve a dimensional reduction, we performed a data-driven principal component analysis (PCA) using all target regions for each of the two biomarkers. The PCA comprised two principal components for [^18^F]PI-2620 target regions (Kaiser Mayer Olkin criteria: 0.805, Bartlett’s test on sphericity: *p* < 0.001; accounted variance for the two components: 72.38%, 11.15%; Fig. 3a, b, Supplementary Table 4) and one principal component for DaT-SPECT target regions (Kaiser Mayer Olkin criteria: 0.829, Bartlett’s test on sphericity: *p* < 0.001, accounted variance for the component: 88.2%; Supplementary Table 5). Principal component 1 of tau-PET (nigrostriatal pathway regions) was associated in the multiple regression analysis with the principal component of DaT-SPECT using age, gender and center as covariates (β =  − 0.442, *p* = 0.015, Durbin-Watson statistics = 2.270; Fig. 3c). There was no association between principal component 2 of tau-PET (dentate nucleus and dorsal midbrain) and the principal component of DaT-SPECT (β = 0.105, *p* = 0.572, Durbin-Watson statistics = 2.084; Fig. 3c).

### Preserved DaT availability relative to tau burden suggests concept of a motor reserve in patients with 4RT

**Fig. 4:**
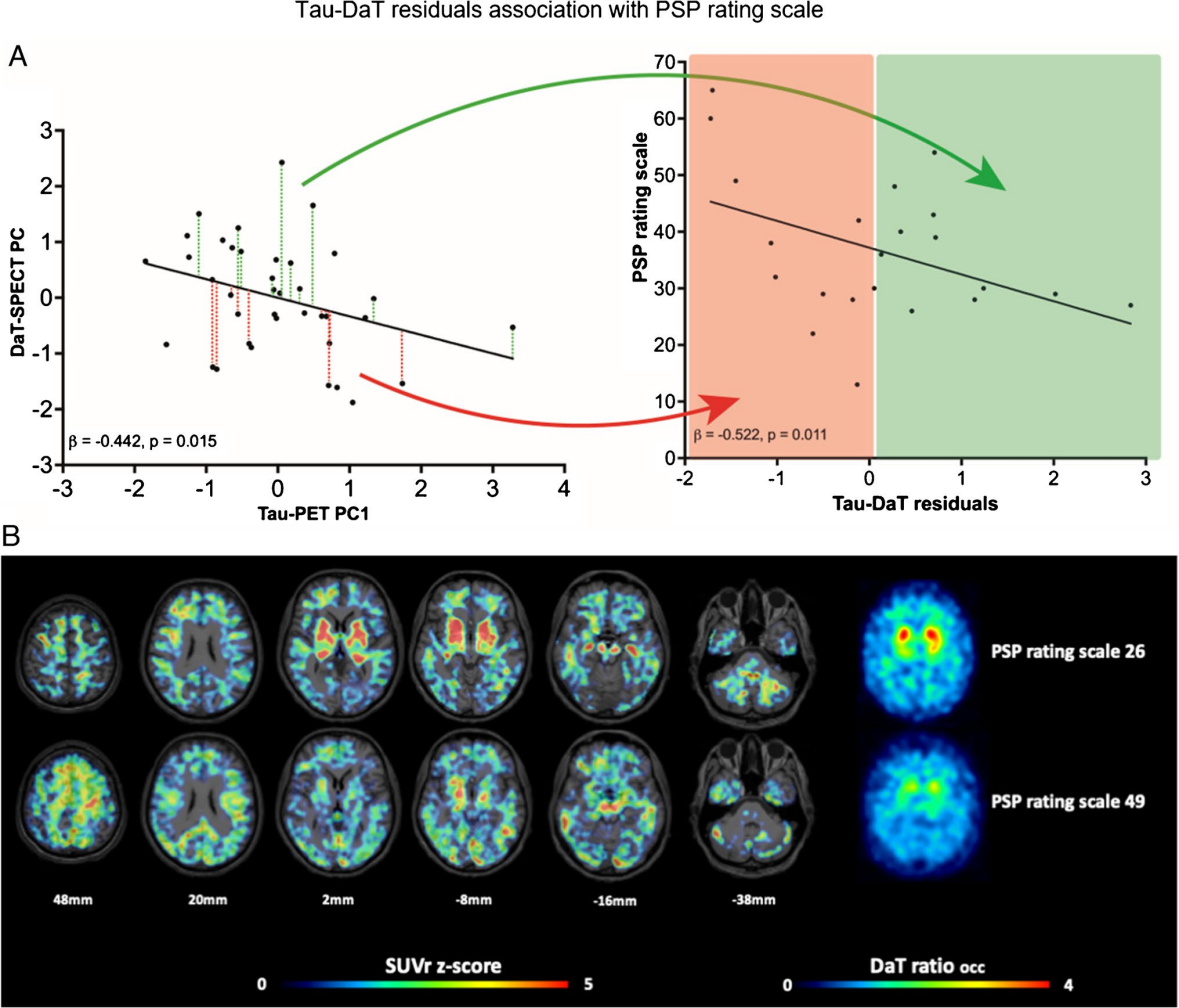
Preserved DaT availability relative to tau burden suggests concept of a motor reserve in patients with 4RT. **A** Residuals of the linear regression between the tau-PET principal component 1 (tau-PET PC1) and the DaT-SPECT principal component (DaT-SPECT PC) positive (green) and negative (red) were obtained as an index of preserved DaT availability despite tau burden. Preserved DaT availability was associated with lower disease severity in the PSP rating scale (right panel). Only patients with PSP rating scale scores are depicted. **B** Exemplary patients with 4R-tauopathies showing high tau burden, preserved DaT availability and mild clinical severity (upper row, 73y, female, PSP-CBS, PSP rating scale: 26) as well as moderate tau burden, strongly decreased DaT availability and severe clinical deterioration (lower row, 79y, female, PSP-CBS, PSP rating scale: 49). Axial slices show individual tau-PET z-scores on a standard MRI-template in contrast against healthy controls and individual DaT SPECT ratio images

**Fig. 5:**
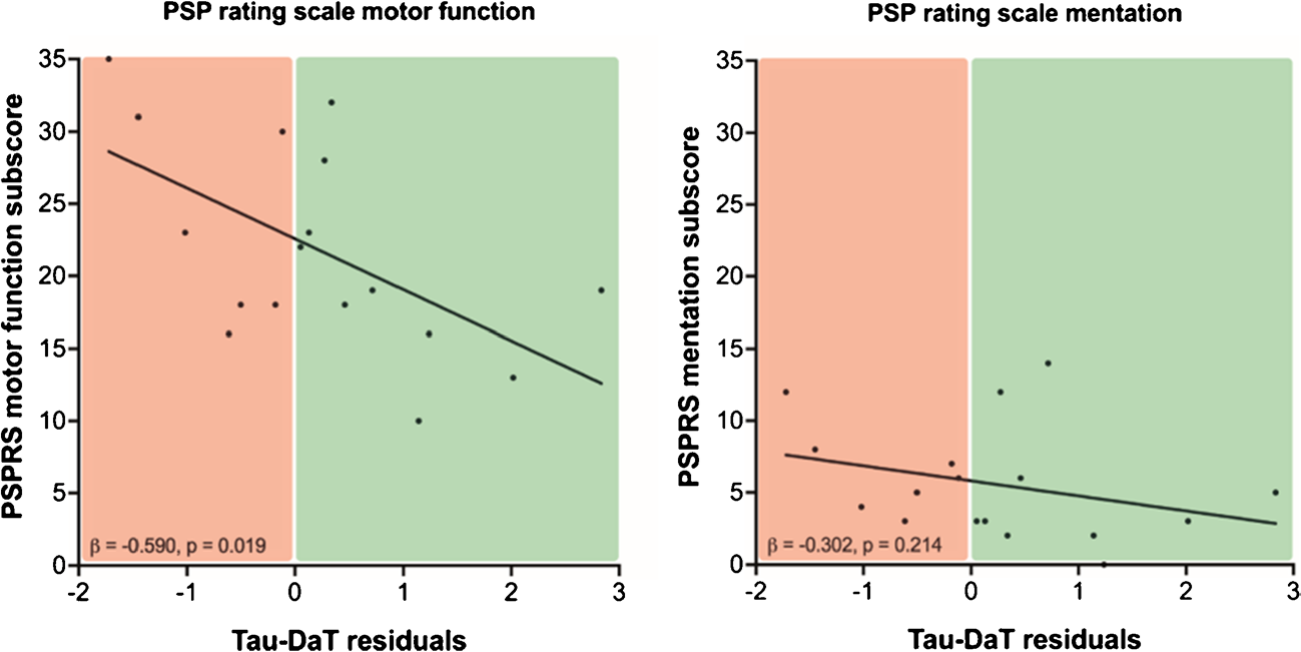
Comparison PSP rating scale items for motor function and mentation. Preserved DaT availability was associated with lower disease severity in the PSP rating scale scores for motor function (left panel), while there was no significant association between preserved DaT availability and lower disease severity in the PSP rating scale scores for mentation (right panel). Only patients with PSP rating scale scores for motor function and mentation items are depicted

Finally, we explored the associations between both biomarkers and clinical severity in patients with 4R-tauopathies. The principal components 1 and 2 of tau-PET did not indicate an association with the PSP rating (PC1: β =  − 0.016, *p* = 0.944, Durbin-Watson statistics = 2.492/ PC2: β =  − 0.190, *p* = 0.401, Durbin-Watson statistics = 2.491), whereas the principal component of DaT-SPECT showed a significant negative association with the PSP rating scale in a multiple regression analysis with age, gender and center as covariates (β =  − 0.512, *p* = 0.013, Durbin-Watson statistics = 2.877). To investigate potential reserve mechanisms, we tested for an association between the residuals resulting from the regression analysis of tau-PET and DaT-SPECT with the PSP rating scale, PSP rating scale items for motor function and items for mentation as well as the Montreal Cognitive Assessment (MOCA) score separately (Supplementary Table 6). PSP rating scale single item scores as well as MOCA scores were only available for patients from Munich. Interestingly, we observed a significant negative association between the residuals resulting from the tau-PET/DaT-SPECT association and the PSP rating scale in the multiple regression (β = -0.522, *p* = 0.011, Durbin-Watson statistics = 2.663; Fig. 4a). Preserved clinical performance was also observed in individual patients with 4R-tauopathies and high tau burden but sustained dopamine transporter availability (Fig. 4b), speaking for a variable vulnerability of dopaminergic neurons in presence of 4R tau. Furthermore, the regression model between the residuals and PSP rating scale motor function items showed an even stronger significant association (β =  − 0.590, *p* = 0.019, Durbin-Watson statistics = 2.319; Fig. 5), whereas items that represent mentation or the Montreal Cognitive Assessment Score did not provide a significant regression model (Mentation items: β =  − 0.302, *p* = 0.214, Durbin-Watson statistics = 2.070; Fig. 5/ MOCA: β = 0.180, *p* = 0.408, Durbin-Watson statistics = 1.392).

To analyze which factor, tau burden, DaT loss or the combination of both, is the best regressor to explain symptoms of the PSP rating scale subsets, we performed a two-way interaction multiple regression analysis. The strongest significant regression factor for motor function items was accounted by the principal component of DaT-SPECT (Supplementary Table 7).

## Discussion

We present the first study using the second generation tau-PET tracer [^18^F]PI-2620 to investigate associations between tau pathology and dopaminergic loss in the striatum of patients with 4R-tauopathies. We demonstrate in vivo that a high tau burden in brain regions involved in direct or indirect pathways of the basal ganglia of patients with 4R-tauopathies is associated with aggravated loss of DaT availability in the striatum as visualized by [^123^I]-Ioflupane SPECT, supporting the role of tau pathology as a potential driver for dopaminergic dysfunction. Regarding dopaminergic functioning, our data show that preserved DaT-SPECT binding relative to the individual tau load was associated with better clinical performance. This highlights the potential of combined tau-PET and DaT-SPECT imaging to detect patients with a high motor reserve due to sustained dopamingeric transmission relative to 4R tau burden.

4R-tau is the key neuropathological feature in PSP and CBD which both show a broad spectrum of cognitive and motor symptoms [24, 28]. *Post mortem* studies revealed that distribution and burden of tau pathology is closely related to different phenotypical subtypes [4, 28, 29]. In this regard, first generation tau-PET tracers such as [^18^F]AV-1451 showed a good correlation between autopsy confirmed cases and of tracer uptake [30–32]. In a recent post mortem tracer binding study of second generation tau tracers [^18^F]PI-2620, [^18^F]MK-6240 and [^18^F]RO-948 in AD, PSP and CBD patients all tracers showed similar binding patterns in AD while only [^18^F]PI-2620 showed a high specificity for PSP and CBD tau pathology [33] highlighting its diagnostic utility for detecting 4R-tau. In addition, a recent in silico study confirms that among the second-generation tau-tracers PI-2620, PM-BB3 and CBD-2115 bind to 4R-tau [34]. [^18^F]PM-PBB3 also showed high binding affinity to 4R-tau but binding in clinically diagnosed patients with MSA [35]. So far, the availbility of PET-tracers binding to 4R-tau is very limited and further development and utilization of specific 4R-tau PET tracers are warranted.

Regarding the regional associations of tau-PET signal in typical target regions of tau accumulation in 4R-tauopathies and striatal DaT binding, we found that only regions of the nigrostriatal pathway and regions involved in direct or indirect pathways of the basal ganglia revealed a significant negative correlation between tau-PET and DaT-SPECT (Fig. 2). The close association between tau deposition within the dopaminergic system and its loss of function supports the view that tau related toxicity reacts as a driver in the degeneration/dysfunction of dopaminergic cells. Second, the correlation of tau deposition within a functionally highly connected system and its loss of function strengthens the concept of interneuronal tau propagation which states that tau is transmitted via synaptic and extrasynaptic pathways while the transmission is enhanced by neuronal activity [12, 36–38]. This concept was also underpinned by our recent investigation that could demonstrate an association between in vivo and ex vivo tau deposition patterns and functional connectivity in a PSP and CBD cohort using [^18^F]PI-2620 tau-PET, histopathology and fMRI [39]. In the current study, the specificity of tau-PET to DaT-SPECT correlations to connected brain regions was determined by lacking associations between tau burden in the dentate nucleus and striatal DaT availability. In this regard, the dentate nucleus is known to be affected in later disease stages of PSP [7] and not considered as a part of the dopaminergic system or the basal ganglia pathway, thus providing value as a negative control region. Furthermore, the PCA analysis revealed that condensed tau-PET signals of the dorsal midbrain and the dentate nucleus showed no correlation with the DaT component while the condensed component of tau signals in functionally connected brain regions of the nigrostriatal pathway showed a significant correlation to the DaT component. This supports that tau spreading in 4R-tauopathies occurs within highly connected brain areas [39] where tau accumulation disturbes neuronal function, such as dopamine transmission (Fig. 3).

Earlier studies using the first generation tau tracers [^18^F]AV-1451 and [^18^F]THK-5351 as well as a recent study using [^18^F]PM-PBB3 demonstrated tau-PET signals in PSP patients correlated with clinical severity [40, 41]. Like in our previous investigations, regional [^18^F]PI-2620 tau-PET signals as well as condensed tau-PET components resulting from the PCA did not correlate with symptom severity measured by the PSP rating scale. Contrary, the DaT component of the PCA showed a significant negative correlation with PSP rating scale scores. This strengthens DaT-SPECT imaging as an index of clinical functionality in 4R-tauopathies while [^18^F]PI-2620 tau-PET imaging preferably serves as a diagnostic tool. More importantly, residuals of the tau-DaT association correlated with clinical perfomance measured by PSP rating scale. We note that the PSP rating scale also contains several non-motor items (see Supplementary Table 6), which implies that the observed resilience is not entirely specific to motor function. To adress this issue, we expanded our analysis on the group of the patients from Munich by separating PSP rating scale motor items and mentation items as well as adding the MOCA scores as an additional measure of cognitive function. Interestingly, this analysis revealed an even stronger association between the residuals of the tau-DaT association and motor symptoms while there was no association to PSP rating scale mentation items or the MOCA scores. These findings support the emerging concept of motor reserve in analogy to the well studied concept of cognitive reserve in AD research [42–44] which suggests that patients with a high motor reserve are able to sustain motor function despite brain neuropathology in target regions [45]. While the concept of motor reserve is being established in the field of PD and the underlying resilience mechanisms remain poorly understood, our data suggest that this concept may also be applicable to 4R-tauopathies. Patients with a high motor reserve could be identified by combined [^18^F]PI-2620 tau-PET and DaT-SPECT imaging, facilitating investigation of possible resilience mechanisms.

While pathological aggregation of 4R-tau is thought to be the key feature in 4R-tauopathies, the potentially crucial role of iron dysregulation needs to be kept in mind [46–48]. Subcortical iron accumulation in PSP is well-documented [49–51] and in Parkinsons disease, the excessive iron accumulation in the substantia nigra is suggested to have toxic effects on the dopaminergic neurons, directly interfering with dopamine synthesis and function [48, 52–54]. Therefore, iron accumulation could possibly have a direct or additional influence on the observed effects of DaT-SPECT and PI-2620 Tau-PET associations in our study. Hence, in future imaging studies, the use of additional iron-sensitive MRI scans should be considered when looking at the tau-/DaT interconnection. Generally, further research in this field is needed to elucidate the precise mechanisms by which iron contributes to tau pathology as it would be of great interest to unravel whether iron dysregulation precedes or coincides with tau aggregation and how they possibly exacerbate each other.

For interpretion of the results of this study, the limited sample size of the cohort consisting of 38 clinically diagnosed PSP/CBS patients and 15 clinically diagnosed α-synucleinopathy disease controls that did not receive autopsy confirmation needs to be acknowledged. However, our data build the grounds for future studies with larger cohorts investigating the association between 4R-tau and the dopaminergic system. Furthermore, there is an ongoing debate whether semiquantitative [^123^I]-Ioflupane SPECT is actually a function of the striatal dopaminergic cell count, i.e. the actual cell loss or if it really reflects axonal dysfunction/DaT density. In a recent study, the postmortem count of substantia nigra pars compacta neurons did not correlate with antemortem DaT binding quantified by DaT-SPECT imaging in 11 confirmed PD cases [55], suggesting that DaT imaging reflects a biomarker of dopaminergic functioning. On the other hand, there is contrary postmortem evidence that reduced striatal [^123^I]-Ioflupane SPECT signals correlate with reduced density of dopaminergic neurons in the substantia nigra in a cohort of 21 patients (12 PD, 4 AD, 7 DLB) [56]. In accordance, another study revealed that antemortem striatal DaT-SPECT binding correlated with the postmortem neuronal cell count of the SN in a cohort of 6 patients (1 PD, 2 DLB, 1 MSA, 1 AD, 1 Creutzfeldt-Jakob) proposing that DaT imaging is a biomarker for nigrostriatal degeneration [57]. Considering the results of our present study, these inconsistent findings lead to the conclusion that the [^18^F]PI-2620 tau-PET signal could be either correlated to reduced dopaminergic function or to nigrostriatal cell loss.

Possible off-target binding also needs to be considered as another limitation of this study. As a derivative of AV-1451 which is known to show several off-target binding sites including the basal ganglia or neuromelanin and iron depositions [58, 59], this issue is especially important for PI-2620. Obviously, second generation tau tracers such as PI-2620 were designed explicitly to adress this problem and so far PI-2620 has shown very high affinity especially to 4R-tau with fast washout from cortical and subcortical areas with substantially lower off-target binding to sites like the basal ganglia, choroid plexus, meninges and other sites commonly found in first generation tau tracers [60, 61]. Especially off-target binding in the basal ganglia, mainly caused by binding to monoamine oxidase, in first generation tau-tracers limited their use in 4R-tauopathies [62, 63]. However for PI-2620, several studies [33, 64, 65] could reveal that this issue has been overcome, which makes us confident that the influence of possible off-target binding in this brain region is limited in this study. However, among all second generation tau-PET tracers, [^18^F]PI-2620 shows off-target binding to neuromelanin which serves as a possible confounder of PET signals in the substantia nigra [61].

Furthermore, during the disease course of 4R-tauopathies, progressive atrophy in regions like thalamus, midbrain and basal ganglia occurs frequently [66] which could potentially mask the PET signal in these regions. We cannot exclude a possible influence of partial volume effects in this study since we did not have a high resolution 3D T1 MRI data set available for all patients, which in consequence would have further restricted our moderate sample size. Indeed increasing atrophy in target regions like the globus pallidus leading to partial volume effects could produce a decrease in tracer signal, especially in patients with long disease duration. Hence, further studies applying partial volume correction will be needed to adress this issue.

In regard to the sometimes rapidly progressing 4R tauopathies PSP and CBS, the relatively long time gap of 3 ± 5 months between DaT and tau imaging also needs to be considered regarding possible limitations of this study as in some cases, disease severity might have progressed between scans.

## Conclusions

Our study suggests that the degree of pathological tau accumulation is associated with dopaminergic dysfunction in 4R-tauopathies and supports the concept of tau being a potential driver of neuronal dysfunction and death in 4R-tauopathies. Furthermore, our data imply that besides the effects of tau on the dopaminergic system, resilience factors may have a major influence on symptom severity emphasizing the potential of combined tau-PET and DaT-SPECT imaging in motor reserve research.

### Supplementary Information

Below is the link to the electronic supplementary material.

Supplementary file1 (DOCX 169 KB)

## Data Availability

Relevant data generated or analyzed during this study are included in this published article and its supplementary information files. Further datasets used and/or analyzed during the current study are available from the corresponding author on reasonable request.
