# Supplementary material for: Tau accumulation is associated with dopamine deficiency in vivo in four-repeat tauopathies
Source: Eur J Nucl Med Mol Imaging. 2024 Feb 17;51(7):1909–22. doi: 10.1007/s00259-024-06637-6 (PMC11139736; doi:10.1007/s00259-024-06637-6)
Supplement: Supplementary file 1 — Supplementary file1 (DOCX 169 KB) [file 259_2024_6637_MOESM1_ESM.docx]

**Supplementary Information**

**Tau accumulation is associated with dopamine deficiency in vivo in four-repeat tauopathies**

Christian Ferschmann^1*^, Konstantin Messerschmidt^4*^ (MD), Johannes Gnörich^1^ (MD), Henryk Barthel^4^ (MD, PhD), Ken Marek^5,6^ (MD), Carla Palleis^2,3,7^ (MD), Sabrina Katzdobler^7^ (MD), Anna Stockbauer^7^ (MD), Urban Fietzek^7^ (MD), Anika Finze^1^, Gloria Biechele^1,8^ (MD), Jost-Julian Rumpf^9^ (MD), Dorothee Saur^9^ (MD), Matthias L. Schroeter^10,11,12^ (MD, PhD, MA), Michael Rullmann^4^ (PhD), Leonie Beyer^1^ (MD), Florian Eckenweber^1^ (MD), Stephan Wall^1^, Andreas Schildan^4^ (PhD), Marianne Patt^4^ (PhD), Andrew Stephens^13^ (MD, PhD), Joseph Classen^9^ (MD), Peter Bartenstein^1,2^ (MD), John Seibyl^5,6^ (MD), Nicolai Franzmeier^2,14^ (PhD), Johannes Levin^2,3,7^ (MD), Günter U. Höglinger^3,7^ (MD), Osama Sabri^4^ (MD, PhD), Matthias Brendel^1,2,3^ (MD, MHBA), Maximilian Scheifele^1^ (MD) for the German Imaging Initiative for Tauopathies (GII4T)^#^

^1^Department of Nuclear Medicine, LMU University Hospital, LMU Munich, Munich, Germany

^2^Munich Cluster for Systems Neurology (SyNergy), Munich, Germany

^3^German Center for Neurodegenerative Diseases (DZNE), Site Munich, Germany

^4^Department of Nuclear Medicine, University Hospital Leipzig, Leipzig, Germany

^5^InviCRO, LLC, Boston, MA, United States of America

^6^Molecular Neuroimaging, A Division of inviCRO, New Haven, CT, United States of America

^7^Department of Neurology, LMU University Hospital, LMU Munich, Munich, Germany

^8^Department of Radiology, LMU University Hospital, LMU Munich, Munich, Germany

^9^Department of Neurology, University Hospital Leipzig, Leipzig, Germany

^10^Clinic for Cognitive Neurology, University Hospital Leipzig, Leipzig, Germany

^11^LIFE - Leipzig Research Center for Civilization Diseases, University of Leipzig, Leipzig, Germany

^12^Max Planck Institute for Human Cognitive and Brain Sciences, Leipzig, Germany

^13^Life Molecular Imaging GmbH, Berlin, Germany

^14^Institute for Stroke and Dementia Research, LMU University Hospital, LMU Munich, Munich, Germany

*contributed equally

**Corresponding author:**

Dr. med. Maximilian Scheifele

Department of Nuclear Medicine

LMU University Hospital, Munich, Germany

Phone: +49 (0) 89 4400 74646

E-Mail: Maximilian.Scheifele@med.uni-muenchen.de

**Supplemental Table 1**

| **Study group** | **4RT** | **α-syn** | **Healthy controls** |
| --- | --- | --- | --- |
| n | 38 | 15 | 23 |
| Age (y) | 69.0 ± 8.5 | 66.1 ± 10.3 | 65.3 ± 9.1 |
| Gender | 17 female / 21 male | 7 female / 8 male | 15 female / 8 male |
| Center | MUC: 25/ LPZ: 9/  MNI: 4 | MUC: 13/  LPZ: 2 | MUC: 10/ MNI: 8/  AUS: 5 |
| PSP Rating Scale | 37.0 ± 12.4 | 16.0 ± 4.2 | n.a. |
| Disease Duration (m) | 26.2 ± 16.6 | 15.6 ± 10.6 | n.a. |
| UPDRS | 36.0 ± 15.5 | 23.3 ± 9.9 | n.a. |

**Supplemental Table 1:** Demographics of main groups of the study. 4RT = 4R-tauopathies; α-syn = probable α-synucleinopathies; n = sample size; n.a. = not available; MUC = scan site Munich, Germany; LPZ = scan site Leipzig, Germany; MNI = scan site New Haven, US; AUS = scan site Melbourne, Australia; UPDRS = Unified Parkinson’s Disease Rating Scale

**Supplemental Table 2**

| **Tau-PET results (SUVr)** | | **4RT** | | **α-syn** | **Healthy controls** |
| --- | --- | --- | --- | --- | --- |
| Putamen right | mean | 1.24 ± 0.17 | | 1.14 ± 0.14 | 1.11 ± 0.10 |
|  |  | p_α-syn_: 0.07 | p_HC_: 0.01 | p_HC_: 0.85 |  |
|  |  | η^2^_α-syn_: 0.07 | η^2^_HC_: 0.10 | η^2^_HC_: 0.001 |  |
| Putamen left | mean | 1.25 ± 0.17 | | 1.14 ± 0.14 | 1.12 ± 0.11 |
|  |  | p_α-syn_: 0.04 | p_HC_: 0.009 | p_HC_: 0.86 |  |
|  |  | η^2^_α-syn_: 0.08 | η^2^_HC_: 0.12 | η^2^_HC_: 0.001 |  |
| Globus pallidus externus right | mean | 1.38 ± 0.22 | | 1.22 ± 0.15 | 1.20 ± 0.12 |
|  |  | p_α-syn_: 0.02 | p_HC_: 0.004 | p_HC_: 0.85 |  |
|  |  | η^2^_α-syn_: 0.10 | η^2^_HC_: 0.14 | η^2^_HC_: 0.001 |  |
| Globus pallidus externus left | mean | 1.32 ± 0.20 | | 1.16 ± 0.13 | 1.18 ± 0.12 |
|  |  | p_α-syn_: 0.01 | p_HC_: 0.01 | p_HC_: 0.63 |  |
|  |  | η^2^_α-syn_: 0.13 | η^2^_HC_: 0.11 | η^2^_HC_: 0.007 |  |
| Globus pallidus internus right | mean | 1.47 ± 0.27 | | 1.27 ± 0.14 | 1.26 ± 0.12 |
|  |  | p_α-syn_: 0.03 | p_HC_: 0.005 | p_HC_: 0.99 |  |
|  |  | η^2^_α-syn_: 0.10 | η^2^_HC_: 0.14 | η^2^_HC_: 0.000 |  |
| Globus pallidus internus left | mean | 1.40 ± 0.21 | | 1.21 ± 0.12 | 1.23 ± 0.13 |
|  |  | p_α-syn_: 0.005 | p_HC_: 0.005 | p_HC_: 0.35 |  |
|  |  | η^2^_α-syn_: 0.16 | η^2^_HC_: 0.13 | η^2^_HC_: 0.03 |  |
| Subthalamic nucleus right | mean | 1.29 ± 0.17 | | 1.16 ± 0.11 | 1.21 ± 0.13 |
|  |  | p_α-syn_: 0.02 | p_HC_: 0.21 | p_HC_: 0.07 |  |
|  |  | η^2^_α-syn_: 0.11 | η^2^_HC_: 0.03 | η^2^_HC_: 0.09 |  |
| Subthalamic nucleus left | mean | 1.29 ± 0.16 | | 1.19 ± 0.10 | 1.19 ± 0.9 |
|  |  | p_α-syn_: 0.03 | p_HC_: 0.03 | p_HC_: 0.79 |  |
|  |  | η^2^_α-syn_: 0.09 | η^2^_HC_: 0.08 | η^2^_HC_: 0.002 |  |
| Substantia nigra right | mean | 1.36 ± 0.20 | | 1.24 ± 0.11 | 1.32 ± 0.12 |
|  |  | p_α-syn_: 0.04 | p_HC_: 0.50 | p_HC_: 0.06 |  |
|  |  | η^2^_α-syn_: 0.09 | η^2^_HC_: 0.008 | η^2^_HC_: 0.11 |  |
| Substantia nigra left | mean | 1.38 ± 0.19 | | 1.28 ± 0.10 | 1.32 ± 0.10 |
|  |  | p_α-syn_: 0.07 | p_HC_: 0.20 | p_HC_: 0.31 |  |
|  |  | η^2^_α-syn_: 0.07 | η^2^_HC_: 0.03 | η^2^_HC_: 0.03 |  |
| Dorsal midbrain | mean | 1.02 ± 0.11 | | 1.00 ± 0.09 | 1.03 ± 0.12 |
|  |  | p_α-syn_: 0.57 | p_HC_: 0.80 | p_HC_: 0.52 |  |
|  |  | η^2^_α-syn_: 0.007 | η^2^_HC_: 0.001 | η^2^_HC_: 0.01 |  |
| Dentate nucleus right | mean | 1.31 ± 0.15 | | 1.21 ± 0.10 | 1.27 ± 0.09 |
|  |  | p_α-syn_: 0.04 | p_HC_: 0.17 | p_HC_: 0.04 |  |
|  |  | η^2^_α-syn_: 0.09 | η^2^_HC_: 0.03 | η^2^_HC_: 0.12 |  |
| Dentate nucleus left | mean | 1.29 ± 0.14 | | 1.19 ± 0.11 | 1.25 ± 0.08 |
|  |  | p_α-syn_: 0.04 | p_HC_: 0.23 | p_HC_: 0.05 |  |
|  |  | η^2^_α-syn_: 0.09 | η^2^_HC_: 0.03 | η^2^_HC_: 0.12 |  |

**Supplemental Table 2:** Group comparison of tau-PET quantification by one-way ANCOVA (age, gender, center as covariates). Partial eta squared shows effect sizes.

**Supplemental Table 3**

| **DaT-SPECT results (z-score)** | | **4RT** | | **α-syn** |
| --- | --- | --- | --- | --- |
| Caudate nucleus right | mean | -2.87 ± 1.21 | | -2.20 ± 1.05 |
|  |  | p_α-syn_: 0.08 |  |  |
|  |  | η^2^_α-syn_: 0.06 |  |  |
| Caudate nucleus left | mean | -2.76 ± 1.22 | | -2.36 ± 1.03 |
|  |  | p_α-syn_: 0.22 |  |  |
|  |  | η^2^_α-syn_: 0.03 |  |  |
| Anterior Putamen right | mean | -2.84 ± 1.22 | | -2.35 ± 1.02 |
|  |  | p_α-syn_: 0.18 |  |  |
|  |  | η^2^_α-syn_: 0.04 |  |  |
| Anterior Putamen left | mean | -2.83 ± 1.25 | | -2.51 ± 1.29 |
|  |  | p_α-syn_: 0.31 |  |  |
|  |  | η^2^_α-syn_: 0.02 |  |  |
| Posterior Putamen right | mean | -3.38 ± 1.28 | | -3.07 ± 1.26 |
|  |  | p_α-syn_: 0.52 |  |  |
|  |  | η^2^_α-syn_: 0.009 |  |  |
| Posterior Putamen left | mean | -2.92 ± 1.12 | | -3.04 ± 1.47 |
|  |  | p_α-syn_: 0.88 |  |  |
|  |  | η^2^_α-syn_: 0.001 |  |  |

**Supplemental Table 3:** Group comparison of DaT-SPECT quantification by one-way ANCOVA (age, gender, center as covariates). Partial eta squared shows effect sizes.

**Supplemental Table 4**

| **Component** | **Total** | **% of Variance** | **Cumulative %** |
| --- | --- | --- | --- |
| 1 | 9.41 | 72.38 | 72.38 |
| 2 | 1.45 | 11.15 | 83.52 |
| 3 | 0.69 | 5.34 | 88.86 |
| 4 | 0.51 | 3.92 | 92.78 |
| 5 | 0.31 | 2.37 | 95.15 |
| 6 | 0.23 | 1.79 | 96.94 |
| 7 | 0.13 | 1.02 | 97.96 |
| 8 | 0.12 | 0.90 | 98.86 |
| 9 | 0.06 | 0.44 | 99.29 |
| 10 | 0.04 | 0.28 | 99.57 |
| 11 | 0.03 | 0.23 | 99.80 |
| 12 | 0.02 | 0.15 | 99.95 |
| 13 | 0.01 | 0.05 | 100.0 |

**Supplemental Table 4:** Principal component analysis eigenvalues of tau-PET.

**Supplemental Table 5**

| **Component** | **Total** | **% of Variance** | **Cumulative %** |
| --- | --- | --- | --- |
| 1 | 5.29 | 88.20 | 88.20 |
| 2 | 0.48 | 7.92 | 96.12 |
| 3 | 0.12 | 1.97 | 98.09 |
| 4 | 0.05 | 0.87 | 98.96 |
| 5 | 0.04 | 0.74 | 99.70 |
| 6 | 0.02 | 0.30 | 100.0 |

**Supplemental Table 5:** Principal component analysis eigenvalues of DaT-SPECT.

**Supplemental Table 6**

| **Motor function** | | | **Mentation** |
| --- | --- | --- | --- |
| **Bulbar** | Dysarthria | Disorientation | |
|  | Dysphagia | Bradyphrenia | |
| **Ocular motor** | Voluntary upward command movement | Emotional incontinence | |
|  | Voluntary downward command movement | Grasping/ imitatative/ utilizing behaviour | |
|  | Voluntary left and right command movement |  | |
|  | Eyelid dysfunction |  |  |
| **Limb motor** | Limb rigidity |  |  |
|  | Limb dystonia |  |  |
|  | Finger tapping |  |  |
|  | Toe tapping |  |  |
|  | Apraxia of hand movement |  |  |
|  | Tremor in any part |  |  |
| **Gait and midline** | Neck rigidity or dystonia |  |  |
|  | Arising from chair |  |  |
|  | Gait |  |  |
|  | Postural stability |  |  |
|  | Sitting down |  |  |

**Supplemental Table 6:** PSP rating scale components that represent motor function compared to items for cognitive function.

**Supplemental Table 7**

| **PSPRS subgroup** | **Regression factor 1** | | | **Regression factor 2** | | | **Regression factor 3** | | |  |
| --- | --- | --- | --- | --- | --- | --- | --- | --- | --- | --- |
|  | **Variable** | **β** | **p-value** | **Variable** | **β** | **p-value** | **Variable** | **β** | **p-value** | |
| **Motor** | Tau 1 | -0.462 | 0.839 | DaT | -3.882 | 0.022 | Tau 1* DaT | 1.962 | 0.428 | |
| **Motor** | Tau 2 | -3.024 | 0.180 | DaT | -2.851 | 0.0751 | Tau 2* DaT | 2.525 | 0.123 | |
| **Mentation** | Tau 1 | 0.101 | 0.941 | DaT | -1.087 | 0.245 | Tau 1* DaT | 2.426 | 0.115 | |
| **Mentation** | Tau 2 | 0.608 | 0.669 | DaT | -1.143 | 0.254 | Tau 2* DaT | 1.252 | 0.230 | |

**Supplemental Table 7:** Multiple linear regression models that explain the associations between PSP rating scale subgroups for motor function (Motor), cognitive function (Mentation), tau-PET and DaT-SPECT principal components. Analyzed components: Tau-PET principal component 1 (Tau 1), Tau-PET principal component 2 (Tau 2) and DaT-SPECT principal component (DaT)

**Supplemental Figure 1**

**
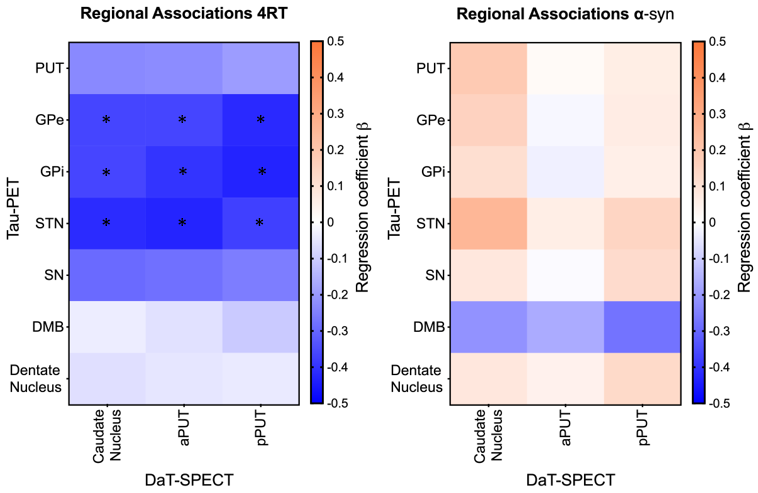
**

**Supplemental Figure 1:**

Blue colors indicate negative multiple regression coefficients β. Orange colors indicate positive multiple regression coefficients β. The analysis of regional associations was performed on the patient´s hemisphere with lower DaT-SPECT mean z-scores. Regions analyzed for tau-PET: putamen (PUT), globus pallidus externus (GPe), globus pallidus internus (GPi), subthalamic nucleus (STN), substantia nigra (SN), dorsal midbrain (DMB) and dentate nucleus (Dentate). Regions analyzed for DaT-SPECT: caudate, anterior putamen (aPUT) and posterior putamen (pPUT).
